# Effects of E-Cigarettes on the Lung and Systemic Metabolome in People with HIV

**DOI:** 10.3390/metabo14080434

**Published:** 2024-08-06

**Authors:** Aline Zaparte, Courtney J. Christopher, Connie Arnold, Lauren Richey, Adairre Castille, Kyle Mistretta, Christopher M. Taylor, Huiyi Lin, Steve Nelson, John P. Kirwan, John W. Apolzan, Shawn R. Campagna, David A. Welsh

**Affiliations:** 1 Department of Internal Medicine, Louisiana State University Health Sciences Center, New Orleans, LA 70112, USA; azapar@lsuhsc.edu (A.Z.); lrich5@lsuhsc.edu (L.R.); acast6@lsuhsc.edu (A.C.); kmistr@lsuhsc.edu (K.M.); snelso1@lsuhsc.edu (S.N.); 2 Department of Chemistry, University of Tennessee, 1420 Circle Drive, Knoxville, TN 37996, USA; cleathe3@vols.utk.edu; 3 Department of Medicine, Louisiana State University Health Sciences, Shreveport, LA 71103, USA; connie.arnold@lsuhs.edu; 4 Department of Microbiology, Immunology, & Parasitology, School of Medicine, Louisiana State University Health Sciences Center, New Orleans, LA 70112, USA; ctay15@lsuhsc.edu; 5 School of Public Health, Louisiana State University Health Sciences Center, New Orleans, LA 70112, USA; hlin1@lsuhsc.edu; 6 Pennington Biomedical Research Center, Louisiana State University System, Baton Rouge, LA 70808, USA; john.kirwan@pbrc.edu; 7 Biological and Small Molecule Mass Spectrometry Core, University of Tennessee, 1420 Circle Drive, Knoxville, TN 37996, USA

**Keywords:** metabolomics, 2,3-Dihydroxypropane-1-sulfonate (DHPS), inflammation, gut metabolome, vaping, neurotransmitters

## Abstract

The popularity of e-cigarettes (vaping) has soared, creating a public health crisis among teens and young adults. Chronic vaping can induce gut inflammation and reduce intestinal barrier function through the production of the proinflammatory molecule hydrogen sulfide (H_2_S). This is particularly concerning for people with HIV (PWH) as they already face impaired immune function and are at a higher risk for metabolic dysregulation, diabetes, and chronic liver disease. Furthermore, PWH experience unhealthy behaviors, making it crucial to understand the systemic metabolic dysregulation and pathophysiological mechanisms associated with vaping in this population. Here, we employed liquid chromatography–mass spectrometry (LC-MS)-based metabolomics to investigate the upper respiratory, circulation, and gut metabolic profiles of PWH who vape (n = 7) and smoke combustible tobacco/marijuana (n = 6) compared to control participants who did not vape or smoke (n = 10). This hypothesis-generating exploratory study revealed systemic alterations in purine, neurotransmitter, and vitamin B metabolisms and tissue-specific changes in inflammatory pathways and cryptic sulfur cycling associated with vaping and combustible tobacco/marijuana smoking in PWH. In addition, this study provides the first link between microbial-derived metabolite 2,3-dihydroxypropane-1-sulfonate (DHPS) and vaping/smoking (tobacco and marijuana)-induced metabolic dyshomeostasis in the gut. These findings highlight the importance of identifying the full biological and clinical significance of the physiological changes and risks associated with vaping.

## 1. Introduction

People with HIV (PWH) experience early onsets of pulmonary comorbidities. The origin of the disparate rates of these comorbidities is multifactorial but includes the high prevalence of unhealthy behaviors such as smoking and other substance use disorders [1,2,3]. While the smoking prevalence among the USA’s general population is 11.5%, in HIV-infected persons this rate is 2–3 times greater [4]. PWH who smoke have an increased risk of smoking-related diseases, including oral infections, pneumonia, chronic obstructive pulmonary disease (COPD), heart diseases and cancer compared to HIV-negative populations [5]. Further, tobacco use is linked to excess mortality in PWH due to non-AIDS-defining illnesses [6].

HIV infection causes chronic immune activation and inflammation, persisting even in individuals on effective antiretroviral therapy (ART). Therefore, PWH exhibit a higher prevalence of metabolic syndrome, characterized by conditions including hypertension, dyslipidemia, and hyperglycemia [7,8,9,10]. In addition, while antiretroviral therapy (ART) is crucial for controlling HIV infection and improving survival, it is linked to several metabolic side effects such as insulin resistance, dyslipidemia, and fat redistribution (lipodystrophy) [8,9]. These adverse metabolic effects can potentially exacerbate the risks associated with vaping.

Although e-cigarettes are promoted as a harmless alternative to smoking, their components may also pose health risks [11,12]. E-cigarettes are battery powered devices that deliver aerosols containing nicotine, artificial flavors and other additives. The ingredients in e-cigarettes vary considerably from product to product but contain recognized carcinogens and toxic substances commonly found in tobacco cigarettes [13]. Propylene glycol, numerous metal and silicate particles, diacetyl (butter flavor), cinnamaldehyde (cinnamon), and benzaldehyde (cherry) are the pulmonary toxicants found at higher concentrations in the e-cigarette aerosols compared to combustible cigarettes [14,15]. Although e-cigarettes may lower the exposure to certain toxicants found in higher concentrations in combustible cigarettes, they are not devoid of harmful substances. The variability in device types, e-liquids, and usage patterns complicates the ability to make definitive statements about the relative levels of all pulmonary toxicants.

More than 13 million people were using these products in 2018, which were produced without adequate quality control or federal regulation [16,17]. The epidemic of e-cigarette or vaping use-associated lung injury (EVALI) in 2019 highlighted the hazards of vaping without an adequate understanding of the biomedical consequences [18]. The acute lung injury was linked to e-liquid contamination with vitamin E acetate and led to thousands of hospitalizations with significant mortality in young, otherwise healthy people [19].

Vaping alters oral and gut microbial composition [20,21]. In primary cultures of human bronchial epithelial cells, e-cigarette liquid shifted the metabolome, which was distinct from changes caused by cigarette smoke condensate [22]. Vaping has been associated with altering the oral microbiome, promoting the growth of pathogenic microbes and increasing risk of infection [20,23]. The oral and gut microbiomes are linked through the oral–gut axis where interorgan microbial and metabolic communication can regulate pathogenesis [24]. Given the link between the oral–gut microbiomes, it is not surprising that vaping has been linked to impaired gut function by contributing to chronic gut inflammation and reduced intestinal tissue integrity, thereby impacting the characteristics of the gut microbiome [25]. This is concerning as the intestinal microbiome occupies a central role as a metabolic “organ” in maintaining overall health [26,27,28].

Additionally, vaping has been reported to alter fatty acid and carnitine metabolism in the urine metabolome [29]. These microbiome and metabolome effects highlight the need to better understand the systemic impact of vaping on the metabolome as metabolites play a vital role in maintaining homeostasis and overall health. Since vaping alters the oral and gut microbiome, which, in turn, may impact the availability of circulating nutrients, and increases the risk of infection, it is vital to understand the systemic impact of vaping on the metabolome, especially in PWH as they face impaired immune function and are at a higher risk for metabolic dysregulation [30]. Thus, the goal of this study was to analyze the systemic impact of e-cigarettes and the smoking of combustibles (tobacco or marijuana) in PWH by analyzing exhaled breath condensate (EBC), oral, and stool metabolomes as well as the availability of nutrients in circulation (plasma and serum metabolomes) and to investigate metabolic markers unique to vaping.

## 2. Materials and Methods

This hypothesis-generating study was designed to explore the systemic metabolomic impact of vaping in PWH. Samples from a subset of participants in the Health Behaviors Syndrome (HBS) Study were assigned into three groups according to e-cigarette use: (1) vaping (with or without smoking combustibles), (2) smoking combustible tobacco or marijuana (no vaping), or (3) controls (no inhaled substance use). Later, the group of participants who smoke combustibles were divided in “tobacco” and “marijuana” cohorts. Participants who smoked both tobacco and marijuana were included in both cohorts (“tobacco” and “marijuana”).

Exhaled breath condensate, oropharyngeal gargle, serum, plasma, and fecal samples were collected for global metabolomics analysis. Unbiased metabolic profiles were generated using liquid chromatography—mass spectrometry and analyzed using MetaboAnalyst 5.0 to identify metabolic alterations in each matrix associated with vaping.

### 2.1. Participant Recruitment

The Louisiana Translational Collaborative on Health Behaviors (LATCH) network is a multi-institutional collaborative established to investigate health behaviors among PWH in Louisiana [31]. Participants for this cross-sectional study were recruited from participants in the HBS Study with testing performed at the Pennington Biomedical Research Center (PBRC) in Baton Rouge and LSU Health in New Orleans. Inclusion criteria were (1) documented HIV-infection; (2) engagement in HIV care; and (3) an age ≥ 18 years. Exclusion criteria for the HBS Study included people with a pacemaker or other implanted electronic device, women who are pregnant, prisoners and cognitively impaired persons. IRB and HIPPA approval and oversight were administered by PBRC using a Central IRB mechanism and informed consent was received from all participants.

### 2.2. Data Collection Instruments

Data collected from interviewer-administered questionnaires include information on demographics, HIV factors, e-cigarette use, tobacco exposure, cannabis/marijuana and alcohol use. Cannabis/marijuana use was measured using the Cannabis Use Disorder Identification Test-Revised (CUDIT-R) [32]. All the data were collected and stored using REDCap^®^. Body measurements were also performed.

### 2.3. Sample Collection

#### 2.3.1. Exhaled Breath Condensate and Oropharyngeal Gargles

Exhaled breath condensation is a non-invasive method to collect exhaled gas samples. EBC samples were collected using RTubes™ (Respiratory Research, Charlottesville, VA, USA), following the manufacturer’s instructions. Briefly, participants breath normally though a cooled RTube™ for ~7 min to collect ~1 mL of EBC. The tubes are transported on ice, aliquoted then immediately stored at −80 °C until the analysis.

To sample the oropharynx, participants vigorously rinsed their mouth then gargled 10 mL of additive-free sterile saline for 30 s. Breaks where permitted with participants holding the saline in the mouth. After 30 s, the solution was expectorated into a sterile specimen collection cup, transported on ice to the laboratory, aliquoted and immediately stored at −80 °C until the analysis.

#### 2.3.2. Serum and Plasma

Whole blood was collected by phlebotomy into BD Vacutainer (Becton-Dickinson, Franklin Lakes, NJ, USA) tubes for serum collection and into lithium heparin-coated or EDTA-coated tubes for plasma. Samples were centrifugated at 1200× *g* for 10 min, aliquoted and stored at −80 °C.

#### 2.3.3. Fecal Material

All participants were provided with a stool collection kit with visual and printed instructions. Fecal samples were frozen at or below −20 °C until processing. Small aliquots were placed in conical tubes and stored at −80 °C until metabolomic analysis.

### 2.4. Metabolite Extractions

All samples were extracted and analyzed at the Biological and Small Molecule Mass Spectrometry Core at the University of Tennessee Knoxville (RRID: SCR_021368). During the metabolomic extraction, samples were kept at 4 °C. Roughly 50 mg of stool and 100 µL of gargle, serum, plasma, and breath condensate were aliquoted for extraction. The exact mass or volume of each sample was recorded and used for sample normalization. Water-soluble metabolites were extracted using an acidic acetonitrile extraction procedure adapted from Rabinowitz and Kimball [33]. Briefly, metabolites from the aliquoted samples were extracted using 1.3 mL of 4:4:2 acetonitrile: methanol: water with 0.1 M of formic acid. All solvents were HPLC grade. Samples mixed with extraction solvent were then kept at −20 °C for 20 min. Following this, the mixture was centrifuged for 5 min, and the supernatant was collected. The remaining sample was re-extracted with 300 µL of extraction solvent. After an additional 20 min at −20 °C and 5 min of centrifugation, the supernatant was collected and combined with the supernatant previously collected. The combined supernatants for each sample were dried under pure nitrogen. Once dried, 300 µL of LC-MS-grade water was added to each sample in preparation for mass spectral analysis.

### 2.5. UHPLC-HRMS

A previously described ultra high-performance liquid chromatography—high resolution mass spectrometry (UHPLC-HRMS) method was used for global metabolomic analysis [34]. Prior to analysis, metabolites were kept at 4 °C in an UltiMate 3000 RS autosampler (Dionex, Sunnyvale, CA, USA). Chromatographic separation was accomplished using a previously described 25 min gradient elution, reverse phase ion-paring method with a water:methanol solvent system, a tributylamine ion pairing reagent, a Synergi 2.6 µm Hydro RP column (100 mm × 2.1 mm, 100 Å; Phenomenex, Torrance, CA, USA), and an UltiMate 3000 pump (Dionex) [35]. The metabolites in the chromatographic eluent were then ionized via negative mode electrospray ionization (ESI) prior to full scan mass spectral analysis with an Exactive Plus Orbitrap mass spectrometer (Thermo Fisher Scientific, Waltham, MA, USA) as previously described [34].

### 2.6. Metabolomics Data Processing

A package from ProteoWizard, msConverter, was used to convert raw mass spectral files to mzML files [36]. All mzML files were imported into an open-source software, metabolomics analysis and visualization engine (El-MAVEN) where metabolites were manually identified using an in-house library based on exact mass (±5 ppm) and retention time (±2 min) [37,38,39]. Metabolite peaks were integrated, and raw peak intensities for identified metabolites were exported from El-MAVEN to a csv file. Prior to statistical analysis, raw spectral data were normalized by either the sample mass or the volume used for extraction.

### 2.7. Statistical Analysis

Either a One-Way Analysis of Variance (ANOVA) followed by Tukey multicomparisons test, Fisher’s test or an Unpaired *t*-test were used on demographics, substance use and HIV data, using the Prism v.9 software. The normalized data were imported into MetaboAnalyst 5.0 and were filtered via the interquartile range (IQR), log transformed, and Pareto scaled prior to statistical analysis [40,41,42]. Partial least squares discriminant analyses (PLS-DAs) were performed in MetaboAnalyst 5.0. For each biological matrix, PLS-DA was used to compare the global metabolomes of control participants, participants who vape, and participants who smoke combustibles (no vaping). Within each biological matrix, PLS-DA pairwise comparisons were used to elucidate the impact of combustible marijuana smoking, combustible tobacco smoking, or vaping on the metabolome by comparing these to the control cohort. Variable importance in projection (VIP) scores were assigned to each metabolite to indicate the importance of each metabolite in contributing to the separation between experimental groups. VIP scores > 1 indicate that a metabolite significantly contributes to the separation of groups in the PLS-DA model based on identified metabolite profiles. Volcano plots were also used to visualize metabolites that were statistically (*p* < 0.1) significantly (fold change > |2|) different between the pairwise comparisons.

## 3. Results

### 3.1. Study Population

Twenty-three participants with HIV were enrolled in this study and divided into control, combustible smoking, and vaping groups. Demographic information and sample characteristics are presented in Table 1, as mean and standard deviation or the absolute number of subjects and the percentage they represent in the group. There was no significant difference regarding the participants’ ages between the three groups [(F(2,18) = 0.7858, *p* = 0.4708]. Participants were predominantly males in all groups. In the control group and in the cohort of participants who smoke combustible tobacco/marijuana, most of the participants reported identifying as African-Americans (70% and 71.42%, respectively). The e-cigarette/vape smoker’s group was 50% African-Americans and 50% Caucasian/White persons. There was no significant difference in the body mass index (BMI) between the groups [(F(2,19) = 2.66, *p* = 0.09].

### 3.2. Substance Use

The summary of substance uses is presented in Table 2; data are represented by mean and standard deviation or the absolute number of subjects and their percentage within the group. The control group reported no vaping or smoking in the past 30 days. Participants who smoke combustible tobacco and marijuana reported an average of 4.83 cigarettes per day, while e-cigarette/vape users reported an average of 6.5 cigarettes per day. The *t*-test revealed no statistical difference between the two groups that reported cigarette use (t(10) = 0.4114, *p* = 0.689). Participants who vape reported an average of 24.33 years of smoking, compared to 16.8 years reported by the participants who smoke combustible tobacco and marijuana; no statistical difference was observed between the groups (t(8) = 0.3873, *p* = 0.701).

All participants in the smoker groups reported lifetime marijuana use, compared to 70% of participants in the control group. Conversely, no one in the control group reported marijuana use in the past 30 days, seven out of the eight participants in the e-cigarette/vape group reported more frequent marijuana use, 21.5 days (SD = 13.47) compared to three out of seven participants in the combustible tobacco/marijuana smokers group that reported using marijuana on 12.67 days (SD = 15.01) in the past 30 days. The Unpaired *t*-test showed no statistical difference between these two groups (t(7) = 0.8968, *p* = 0.3996).

All participants in the smoker groups reported lifetime alcohol use, compared to 75% of participants in the control group. However, a minority in each group had recent alcohol use (in the last 30 days), four people in the control group and two people each in the vape user and smoker groups. Among those that reported alcohol use in the past 30 days, the control group reported 5 days of alcohol use in the last 30 days compared to both smoking groups (both, 8 days). There was no significant difference in the scores for the control group and the other groups [(F(2,4) = 2.571, *p* = 0.1914].

### 3.3. HIV-Related Variables

The average of years since HIV diagnosis in the control group was 16 years (SD = 11.8), 23 years (SD = 8.74) in the combustible tobacco/marijuana group and 16.33 years (SD = 6.53) in the vaping/e-cigarette group; no statistical difference was found between the groups [F(2,19) = 1.062, *p* = 0.3655]. All three groups endorsed high adherence to antiretroviral therapy, with no statistical differences [(F(2,19) = 0.8355, *p* = 0.449]. Clinical laboratory results were only available for 10 participants. The viral loads ranged from <50 copies/mL to undetectable, the mean of T CD4+ was 818.9 cells/μL (SD = 347.19) and the mean of TCD8+ cells was 896.5 cells/μL (SD = 554.59).

### 3.4. Global Metabolomics

Exhaled breath condensate, oropharyngeal gargle, serum, plasma and stool samples were collected from participants and analyzed to identify system-wide vaping-induced changes in the metabolome. EBC was used to non-invasively assess the lung metabolome while oropharyngeal gargle is representative of the oral metabolome. Together, EBC and oropharyngeal gargle provide insights into the upper respiratory metabolome. Serum and plasma samples served to access the metabolites in circulation, and stool samples were used to investigate the gut metabolome. There were 207 metabolites identified based on exact mass and retention time using an in-house standard library. Out of these 207 metabolites, 122 (59%) were detected across all biological matrices. There were 2, 7, and 20 metabolites unique to the upper respiratory, circulating, and gut metabolomes, respectively. Using 3D PLS-DAs, we assessed vaping and smoking-induced shifts in the global metabolomes for each matrix (S1). This revealed that vaping dramatically altered the upper respiratory, circulating, and gut metabolomes.

Across all biological regions analyzed, vaping resulted in more evident metabolome differences than combustible smoking (tobacco/marijuana) based on the extent of separation observed in the 3D PLS-DA models (Figure S1), with the vaping cohort showing complete separation from the control while the combustible smoking cohort had an overlap with the control in the plasma and EBC. Additionally, within each matrix, we individually compared vaping, combustible tobacco smoking, and combustible marijuana smoking to the control cohort in pairwise PLS-DA models in order to (1) identify metabolic perturbations associated with vaping and (2) investigate how vaping-induced metabolome alterations are different from smoking (combustible) tobacco or marijuana by identifying specific metabolites impacted by vaping but not (combustible) smoking. From the “smoking” cohort, further analyses grouped any participant who reported combustible tobacco smoking, regardless of vaping or marijuana use, to the “tobacco” group, and any participant reporting marijuana use, regardless of vaping or tobacco use, to the “marijuana” group. Metabolites from this analysis with a fold change > |1.5| (*p* < 0.1) or a VIP score > 1 were used for further analysis.

#### 3.4.1. Exhaled Breath Condensate Metabolome

**Figure 1:**
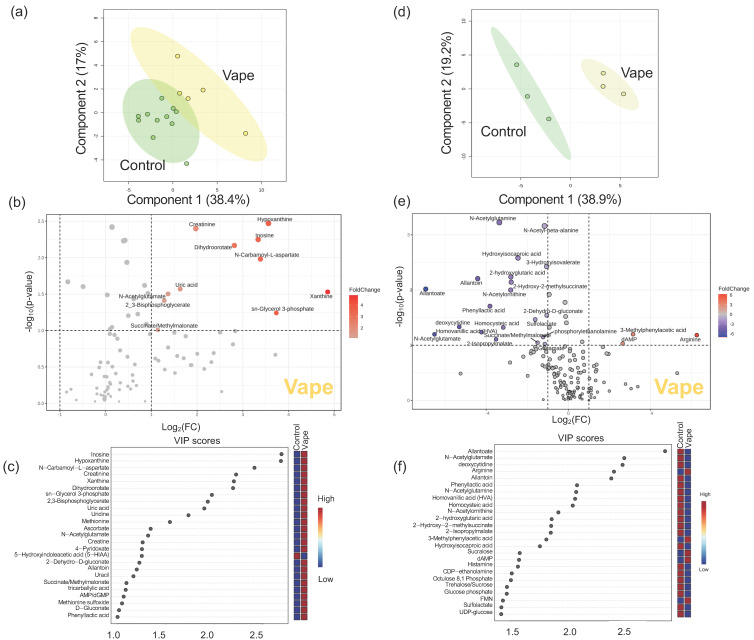
Vaping induces changes in metabolites in the upper respiratory tract. Vaping has a clear impact on exhaled breath condensate and gargle. Partial least squares discriminant analysis (PLS−DA) demonstrates global metabolome differences between participants who vape (yellow) and the control cohort (green) in exhaled breath condensate (EBC) (**a**) and oropharyngeal gargle (**d**). Metabolites altered by vaping in the EBC (**b**) and oropharyngeal gargle (**e**) metabolomes are shown in a volcano plot where the x−axis displays the log_2_ fold change and the y−axis displays significance. The dotted lines represent cutoff values of *p* < 0.1 and fold change > 2 in either direction. Metabolites in red were significantly increased in the participants who vape and decreased in the control cohort. Each metabolite was assigned a variable importance in projection (VIP) score to assess its contribution to the differences in metabolic profiles between participants who vape and the control cohorts. The 25 metabolites with the highest VIP scores in EBC (**c**) and oropharyngeal gargle (**f**) were shown. A VIP score > 1 indicates the metabolite is a significant driver of the observed separation between groups.

**Figure 2:**
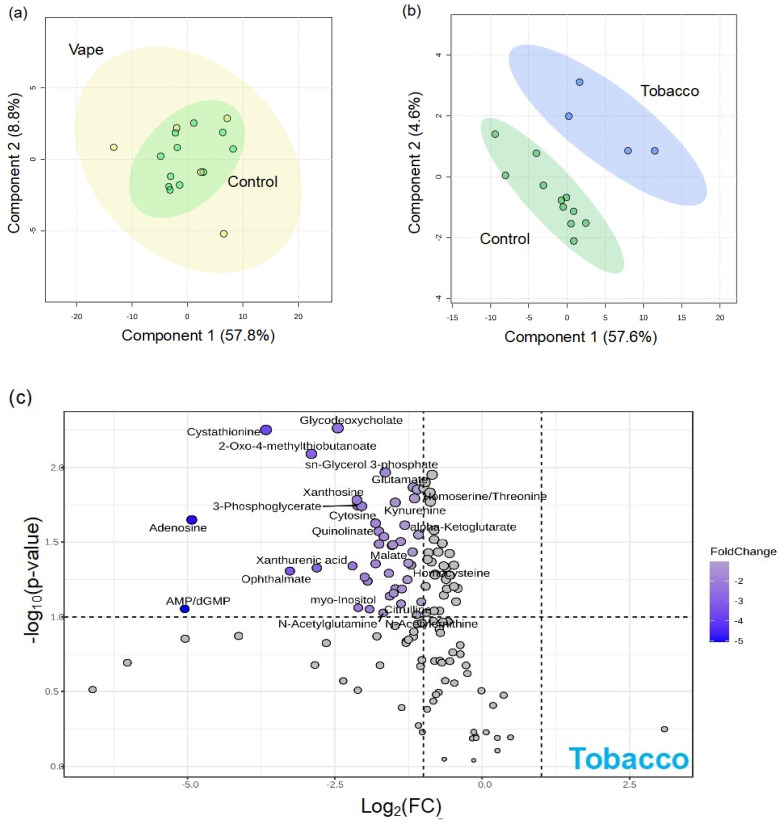
Metabolite comparisons between participants who vape and participants who smoke combustible tobacco in serum samples. Partial Least Squares Discriminant Analyses (PLS−DAs) were used to assess the impact of vaping (yellow) (**a**) combustible tobacco smoking (blue) (**b**) on the serum metabolome by comparing them to the control cohort (green). The volcano plot (**c**) demonstrates metabolites altered by combustible tobacco smoking. The x−axis displays the log_2_ fold change and the y−axis displays significance. The dotted lines represent cutoff values of *p* < 0.1 and fold change > 2 in either direction. Metabolites in blue are significantly increased in the control cohort and decreased in the tobacco cohort.

Within the exhaled breath condensate metabolome, 97 metabolites were identified. The vaping cohort had greater separation from the control cohort than the smoking cohort indicating that vaping altered the metabolome to a greater degree than combustion smoking (Figure S1). Direct comparison of the vaping and control cohort showed 25 (26%) metabolites were impacted by vaping (Figure 1), with 5 metabolites unique to vaping. Remarkably, there were no notable differences observed in the global metabolome profiles of the tobacco/marijuana smoking cohort (Figure S2). Surprisingly, only four metabolites were upregulated in the marijuana cohort and five were upregulated in the tobacco cohort (Figure S2). Metabolites only altered by vaping included uric acid, methionine, 5-hydroxyindoleacetic acid (5-HIAA), methionine sulfoxide, and d-gluconate. Metabolites with the highest VIP scores were inosine (2.7), hypoxanthine (2.6), *N*-carbamoyl-l-aspartate (2.4), creatinine (2.2), and xanthine (2.2) (Figure 2). In summary, in EBC, vaping was associated with the upregulation of creatinine, inosine, xanthine, uric acid, and *N*-acetylglutamate (Figure 2).

#### 3.4.2. Oropharyngeal Metabolome

In the oropharyngeal metabolome, 162 metabolites were identified, and PLS-DA comparing vaping, smoking, and controls showed the complete separation of groups, indicating dramatic differences in the overall metabolic profiles (Figure S1). There were 47 (31%) metabolites significantly altered by vaping, with all being downregulated except arginine, dAMP, and 3-metlylphenyllacetic acid (Figure 1). There were three metabolites only altered in participants who vape: sucralose, glutathione, and riboflavin. Metabolites with the highest VIP scores were allantoate (2.9), N-acetylglutamate (2.5), deoxycytidine (2.5), arginine (2.4), and allantoin (2.4) (Figure 1).

#### 3.4.3. Peripheral Blood Metabolome

To assess how vaping impacts the availability of nutrients in systemic circulation, we examined plasma and serum metabolomes. Plasma samples collected using EDTA (ethylenediaminetetraacetic acid) or lithium heparin as specific anticoagulants can confound results [43]. We analyzed both plasma EDTA and plasma heparin samples. There were 140 identified metabolites in the serum metabolome, yet no notable vaping-related alterations were observed in the global metabolome, as highlighted by the overlap of groups in the PLS-DA (Figure S1). Only one metabolite, guanine, was significantly altered by vaping in the serum metabolome (Figure S3. While vaping had little to no observable differences in identified metabolites of the serum metabolome, combustion smoking provoked significant changes with 59 altered metabolites (Figure 2).

**Figure 3:**
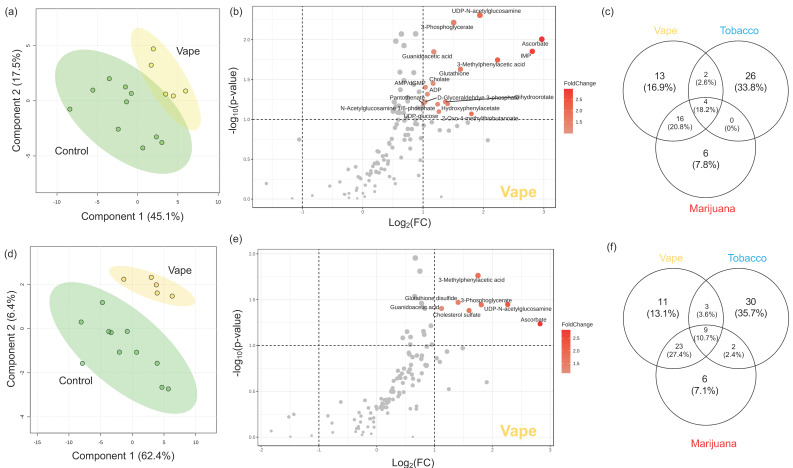
Participants who vape display dramatic metabolome alteration in plasma, according to samples from PWH. Partial Least Squares Discriminant Analyses (PLS−DAs) were used to assess the impact of vaping (yellow) on the global plasma EDTA (**a**) and plasma heparin (**d**) metabolomes by comparing them to the control cohort (green). Volcano plots were used to visualize metabolites altered by vaping in the plasma EDTA (**b**) and plasma heparin (**e**) metabolomes. The x−axis displays the log_2_ fold change and the y−axis displays significance. The dotted lines represent cutoff values of *p* < 0.1 and fold change > 2 in either direction. Metabolites in red are significantly increased in the vaping cohort and decreased in the control cohort. Each metabolite was assigned a variable importance in projection (VIP) score to assess its contribution to the differences in metabolic profiles in the PLS−DA model. A VIP score > 1 indicates that the metabolite is a significant driver of the observed separation between groups, and metabolites with a VIP score > 1 for each pairwise comparison were used to identify metabolites altered only be vaping. The Venn diagram comparing these metabolites shows 13 and 11 unique markers for vaping in plasma EDTA (**c**) and heparin (**f**), respectively.

In the plasma EDTA samples, 129 metabolites were identified. The 3D PLS-DA demonstrates the clear differences in the overall metabolic profiles resulting from vaping (Figure S1). There were 36 differentially abundant metabolites in the vaping cohort (Figure S4). Of these, 13 were altered by vaping but not tobacco/marijuana smoking (Figure 3). Glutathione disulfide, sulfolactate, pyruvate, and pantothenate were among the metabolites unique to vaping. There were 14 metabolites altered by vaping, tobacco, and marijuana smoking including glutathione disulfide, sulfolactate, pyruvate, and pantothenate, among others (Figure 3). Metabolites most responsible for metabolic profile differences between vaping and control cohorts were ascorbate (2.8), IMP (2.3), UDP-*N*-acetylglucosamine (2.1), 3-phosphoglycerate (1.9), and 3-methylphenylacetic acid (1.7) (Figure S4). Overall, vaping was associated with the significant upregulation of cholate, ascorbate, glutathione, and pantothenate (Figure 3).

Greater vaping-related differences in circulating nutrients were observed in heparinized plasma samples where the vaping metabolome more dramatically separated from the control (Figure S1). There were 125 identified metabolites, and 46 (37%) displayed vaping-induced changes. This was the greatest percentage of vaping-altered metabolites out of all matrices analyzed. Of these 46 metabolites, 11 were unique to vaping and 9 were differentially abundant and altered across vaping and tobacco/marijuana smoking cohorts (Figure 3). The metabolites uniquely altered by vaping included glutathione disulfide, phenylalanine, indole, and tryptophan, among others. Similarly, metabolites most substantially driving separation between the vaping and control cohorts based on VIP scores were glutathione disulfide (2.4), ascorbate (2.4), 3-phosphoglycerate (2.3), cholesterol sulfate (2.2), and UDP-*N*-acetylglucosamine (2.1) (Figure S4). Overall, significant upregulations of cholesterol sulfate, glutathione disulfide, and ascorbate were associated with vaping (Figure 3).

#### 3.4.4. Gut Metabolome

**Figure 4:**
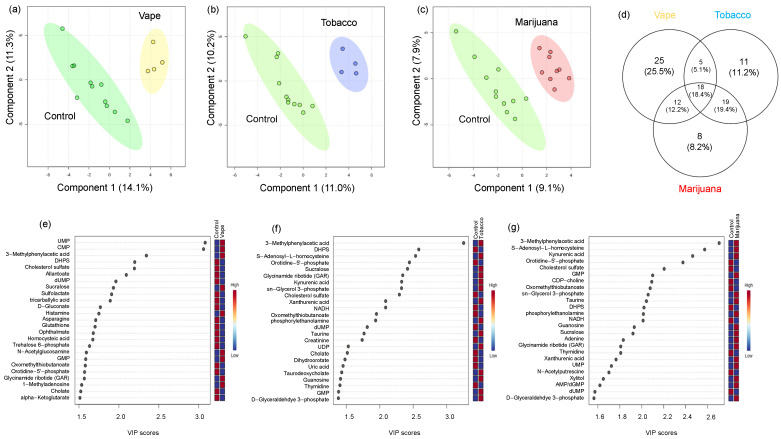
Vaping impacts the metabolic profile more than combustible tobacco or vaping in the stool metabolome. Partial Least Squares Discriminant Analyses (PLS−DAs) were used to assess the impact of (**a**) vaping (yellow), (**b**) combustible tobacco smoking (blue), and (**c**) combustible marijuana smoking (red) on the global metabolome by comparing them to the control cohort (green). Each metabolite was assigned a variable importance in projection (VIP) score to assess its contribution to the differences in metabolic profiles from the PLS-DA models for vaping (**e**), combustible tobacco smoking (**f**), and combustible marijuana smoking (**g**). The 25 metabolites with the highest VIP scores were shown. A VIP score > 1 indicates that the metabolite is a significant driver of the observed separation between groups. Metabolites with a VIP score > 1 for each pairwise comparison were used to identify metabolites altered only be vaping. The Venn diagram (**d**) comparing these metabolites shows 25 unique markers for vaping. (CTRL, control).

**Figure 5:**
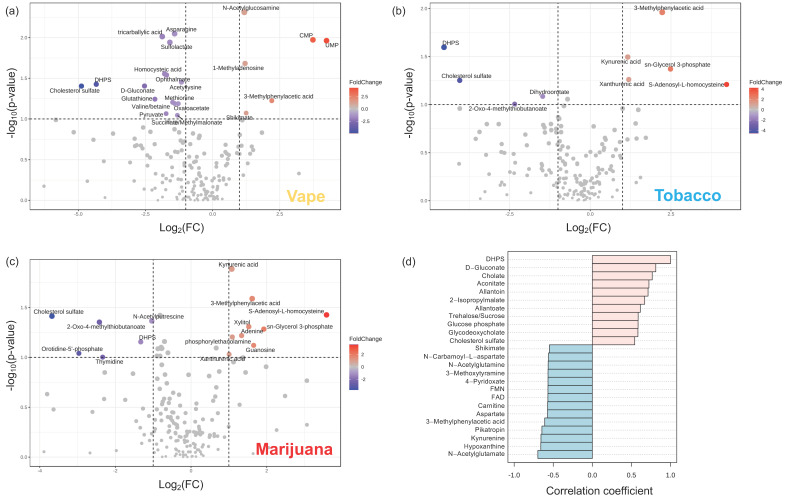
Smoking alters the stool metabolite profile. Metabolites significantly altered by vaping (**a**), combustible tobacco smoking (**b**), and combustible marijuana smoking (**c**) are shown with metabolites in blue significantly increased in the control cohort. The x−axis displays the log_2_ fold change and the y−axis displays significance. The dotted lines represent cutoff values of *p* < 0.1 and fold change > 2 in either direction. Pearson’s r correlation coefficients (**d**) were used to identify metabolites correlated with DHPS in the stool metabolome of participants who vape. DHPS, 2,3−dihydroxypropane−1−sulfonate.

In the fecal metabolome, there were 186 identified metabolites. The PLS-DA model using these metabolites revealed that the stool metabolome in the vaping cohort was clearly distinct from the control cohort as was the smoking (tobacco/marijuana) cohort, although less dramatically (Figure S1). To identify specific vaping-related metabolic alterations, we compared the vaping and control cohorts, and found 60 metabolites (32% of identified metabolites) differed (Figure 4). Of the 60 metabolites impacted by vaping, 25 were unique to vaping, displaying no significant differences in tobacco/marijuana smoker versus control (Figure 5). Sulfolactate, glutathione, indole, shikimate, tryptophan, folate, allantoin, and phenylalanine were among the unique metabolic signatures of vaping. In contrast, 18 small molecules differed from controls in both the vaping and tobacco/marijuana smoking cohorts (Figure 5). These included CMP, 3-methylphenylacetic acid, DHPS (2,3-dihydroxypropane-1-sulfonate), cholesterol sulfate, sucralose, cholate, methionine, 5-methyltetrahydrofolate, and xanthurenic acid, among others. Metabolites with the highest VIP scores between vaping and control cohorts, therefore having a compelling impact on the metabolome differences, were UMP (uridine monophosphate) (3.1), CMP (cytosine monophosphate) (3.1), 3-Methylphenylacetic acid (2.3), and DHPS (2.2) Figure 4). Overall, the stool metabolome revealed a downregulation with vaping of metabolites involved in sulfur metabolism: DHPS, sulfolactate, methionine, glutathione, pyruvate, asparagine, cholesterol sulfate, and an upregulation of *N*-acetylglucosamine, 1-methyladenosine, 3-methylphenylacetic acid, CMP, and UPM (Figure 5).

## 4. Discussion

While vaping has been promoted as a safer alternative to smoking, newer reports indicate that it carries substantial health risks. The impact on respiratory and cardiovascular health [44,45,46], the potential for nicotine addiction [47], the exposure to harmful chemicals [48], and the negative effects on mental health underscore the need for caution [49]. Public health initiatives should continue to address the risks of vaping, especially among vulnerable populations, to mitigate these health impacts [44,50].

### 4.1. Vaping Induces Considerable Changes in the Upper Respiratory Metabolome

Within the oral cavity, EBC and gargle metabolomes were analyzed, and vaping resulted in alterations in both metabolomes, with 26% of identified metabolites altered in EBC and 29% in oropharyngeal gargle. Of the metabolites exhibiting vaping-associated differences in EBC, inosine, hypoxanthine, xanthine, AMP, uric acid and allantoin were all upregulated and significant drivers of metabolome differences resulting from vaping. All these metabolites are involved in purine metabolism. Additionally, creatine and creatinine were also upregulated in participants who vape, and these nitrogen-containing metabolites are synthesized from amino acids. Purines are directly connected to creatine metabolism through protein degradation. The breakdown of protein yields amino acids which are used for the synthesis of nitrogen-containing metabolites, specifically purines and creatine. Other notable nitrogenous metabolites altered by vaping were 4-pyridoxate, a vitamin B_6_ metabolite and cofactor for purine synthesis, and 5-HIAA, a tryptophan catabolite. Upregulation of purines, vitamin B and creatine in the EBC may suggest that vaping induces increased flux in nitrogen metabolism.

Unlike EBC, nearly all statistically significant metabolites altered by vaping in the oropharyneal gargle metabolome were downregulated. Arginine, dAMP, and 3-metlylphenyllacetic acid were downregulated. The amino acid arginine plays a vital role in regulating metabolism throughout the body [51]. For example, it is involved in protein biosynthesis, urea and polyamine metabolism, as well as nitric oxide production. Nitric oxide helps defend against oxidative stress and regulates blood pressure, acting as a vasodilator, and decreases the risk of cardiovascular disease [52]. Increased arginine levels in the vaping cohort may be reflective of decreased conversion to nitric oxide, and decreased nitric oxide leads to increased blood pressure, which is a known physiological impact of vaping [52]. Metabolites downregulated in EBC due to vaping included allantoate and allantoin, catabolic products of uric acid. It is also notable that acetylated metabolites were downregulated since acetylation is a form of epigenetic regulation to redirect cellular metabolism [53].

### 4.2. Availability of Nutrients in Circulation Are Affected by Vaping

Although there were no significant differences in the serum global metabolome profiles of participants who vape, one metabolite, guanine, was significantly altered by vaping in the serum metabolome. Combustible marijuana smoking resulted in no statistically significant metabolic alterations. However, the serum metabolome exhibited vast differences between the control cohort and participants who smoke combustible tobacco, with 42% of metabolites altered. These metabolites were mostly involved in purine, neurotransmitter, and one-carbon metabolism.

In contrast, substantial vaping-induced alterations were observed in plasma (EDTA and heparin) metabolomes with vaping related to clear shifts in purine metabolism as IMP, AMP, and GMP were upregulated. Because the biological matrix has an influence on metabolite availability, serum, plasma EDTA and plasma heparin samples can display different metabolomes [43,54]. Serum has no preservatives and needs fibrinogen for clot formation, whereas anticoagulants can bind to Ca^2+^ and Mg^2+^ (EDTA) or inhibiting thrombin (heparin), influencing enzymes and changing ion concentrations, which can result in different metabolic profiles between the samples [43,54]. However, to gain a global view of vaping-induced alterations in the plasma metabolomes, differentially abundant metabolites from plasma EDTA and plasma heparin were combined.

Additionally, there were clear indicators of altered antioxidant production and capacities in participants who vape. Ascorbate, glutathione, and glutathione disulfide were upregulated in participants who vape, and these metabolites play pivotal roles in defending against oxidative stress. For example, ascorbate is reported to be the most effective aqueous-phase antioxidant in human blood [55]. Glutathione, a tripeptide made from glutamate, cysteine, and glycine, is a powerful antioxidant responsible for maintaining integrity of cellular functions and homeostasis [56,57]. Both vaping and cigarette smoke are known to induce oxidative stress, which is a driving factor in many smoking/vaping-related diseases [58,59,60]. Pantothenate, known as vitamin B_5_, was impacted by vaping, and plays an important role in fatty acid, glucose, fatty acid, and amino acid metabolism [61]. It is also known to encourage the expression of inflammatory cytokines. With vaping known to induce oxidative stress, it is intriguing that antioxidants such as ascorbate, glutathione, and pantothenate are more abundant in the plasma of participants who vape compared to controls. Cholesterol sulfate was also increased in the plasma of participants who vape. This metabolite is involved in cellular membrane stability, signal transduction, and promoting cholesterol biosynthesis [62]. Increased plasma cholesterol sulfate levels often correspond to an array of inflammatory diseases as it modulates arachadonic acid (AA) metabolism, and AA is a proinflammatory molecule [63,64].

### 4.3. Vaping-Induced Metabolic Dyshomeostasis Is Most Evident in the Gut Metabolome

Surprisingly, the most substantial vaping-induced metabolome alterations were observed in the stool, where 32% of identified metabolites were impacted in participants who vape. Similar to the oral metabolome, vaping triggered a profound impact on purine and nitrogen metabolism as CMP, UMP, GMP and histamine were significantly upregulated and allantoate, dUMP, and glutathione were significantly downregulated in the stool metabolome of participants who vape. Other notable metabolites altered by vaping were cholesterol sulfate, DHPS, cholate, methionine, and pyruvate. These results are consistent with the known impact of nicotine on energy homeostasis [65,66].

The data demonstrated a compelling connection between DHPS and metabolic dysregulation as DHPS was a key driving factor (noted by VIP scores) for the observed global metabolome differences between vaping, tobacco, and marijuana smoking compared to controls. This was an unexpected finding as DHPS is a microbial derived metabolite with an unknown role in human physiology, yet it was downregulated in the stool of all substance-use cohorts compared to the control cohort [67]. DHPS can be acquired via the bacterial metabolism of dietary sulfoquinovose (SQ), one of the most abundant organosulfonates. Leafy greens and other photosynthetic plant materials in the diet are rich sources of SQ [68]. Bacterial transformation of SQ and DHPS results in the production of H_2_S [67,68,69,70,71], and this is particularly interesting as H_2_S is a major regulator of cellular functions through both epigenetic and non-epigenetic regulation [72]. At metabolic homeostasis, H_2_S promotes mitochondrial respiratory function, immune regulation, and cellular signaling via acetylation and methylation [72]. However, disruptions in metabolic homeostasis can result in excess H_2_S which promotes oxidative and mitochondrial dysfunction and an impaired intestinal barrier [67,73]. Decreased DHPS in participants who vape/combustible smoke may be indicative of metabolic dyshomeostasis via increased flux through DHPS. This increased flux through DHPS generating excess H_2_S may trigger a complex inflammatory and immune cascade. As it is known that vaping leads to gut inflammation [25] and reduced intestinal barrier integrity, these data could link altered sulfur metabolism to smoking and vaping-induced metabolic dyshomeostasis in the gut through DHPS. This is particularly important for PWH since changes in the gastrointestinal tract can contribute to disease progression and this community already experiences impaired immune functions and a weakened intestinal barrier [74,75].

To gain insight into the potential physiological role of DHPS, correlations using Pearson’s r correlation coefficients were used to identify metabolites correlated with DHPS (Figure 5). This revealed that DHPS was positively correlated with cholate, allantoin, allantoate, trehalose/sucrose, cholesterol sulfate, glucose phosphate, d-gluconate, and glycodeoxycholate (Figure 5). Metabolites with negative correlations to DHPS included shikimate, 4-pyridoxate, FMN, carnitine, kynurenine, hypoxanthine and acetylated amino acids, among others. (Figure 5). Metabolites positively correlated with DHPS, allantoin and allantoate, are related to purine degradation and are intermediates in the conversion of uric acid to urea and ammonia. As previously noted, purine metabolism was profoundly impacted by vaping in the stool metabolome, so it is interesting that DHPS is correlated with these metabolites. Additionally, other metabolites positively correlated with DHPS suggest that DHPS is linked to glucose, cholesterol, and bile acid metabolism. Combined, this may highlight a novel physiological connection between DHPS and purine, glucose, and cholesterol metabolism. In contrast, metabolites that negatively correlated with DHPS are involved in vitamin B, energy, and neurotransmitter metabolism. The metabolite, 4-pyridoxate, is a catabolic product of vitamin B_6_, which is a vital cofactor for neurotransmitter metabolism. Shikimate and kynurenine are produced by microbes in the gut and are involved in tryptophan and neurotransmitter metabolism. Additionally, they are proposed to have immunomodulatory benefits, promote intestinal immune homeostasis, and act as signaling molecules to regulate bacterial and host metabolism [76,77,78,79]. In general, B vitamins serve as cofactors for purine synthesis, one-carbon metabolism, antioxidant defense, neurotransmitter synthesis, and ATP generation [80]. Carnitine is involved in energy metabolism through the beta oxidation of fatty acids to generate acetyl-CoA and ATP. Increased carnitine in the gut can be problematic as microbes degrade carnitine into trimethylamine (TMA) which is oxidized to trimethylamine N-oxide (TMAO) and can promote atherosclerosis [81]. Similarly, FMN is a cofactor for the conversion of vitamin B_12_ to FAD, thereby linking FMN to vitamin B and energy metabolism. Combined, the metabolites negatively correlated with DHPS are broadly involved in energy metabolism, demonstrating a potential physiological role of DHPS in vaping/smoking metabolic dyshomeostasis.

Although the gut microbial community phylogenetics were not assessed in this study, alterations in microbial-derived metabolites like DHPS, shikimate, and kynurenine indicate that vaping alters microbes in the gut. This is supported with previous findings by Whitehead et al. providing evidence that gut microbial composition and diversity are altered by inhaled nicotine in mice [21]. They reported differences in nine genera between nicotine inhalation and controls: *Odoribacter*, *Turicibacter*, *Roseburia*, *Eubacterium*, *Sutterella*, *Desulfovibrio*, *Anaeroplasma*, *Prevotella*, and *Akkermansia*. These findings were of great interest because *Eubacterium* convert dietary SQ to DHPS and *Desulfovibrio* convert DHPS to H_2_S [67]. In addition, this also further implicates DHPS in cholesterol, choline, and carnitine metabolism as *Desulfovibrio* is a known regulator of cholesterol and bile acid metabolism and TMAO production [82,83]. Combined, this data-driven approach based on global metabolomics provides the first link of DHPS to alterations in the gut metabolome in vaping, tobacco, and marijuana use. We hypothesize that DHPS is implicated in dyshomeostasis in sulfur and cholesterol metabolism and propose that it is a missing link in the pathophysiology of inflammation and that it may be a key regulator of inflammation and overall human health. Further studies are warranted to validate this hypothesis as well as to investigate the ramifications of DHPS dyshomeostasis in human physiology and pathophysiology. To the best of our knowledge, we are the first to demonstrate this novel association between DHPS and vaping-induced metabolic dysregulation, suggesting cryptic sulfur metabolism may play a role in pathophysiological outcomes associated with vaping.

### 4.4. Vaping Leads to Systemic Metabolome Alterations as Well as Distinctive Metabolic Patterns Not Observed in Tobacco or Marijuana Smoking

Vaping resulted in a systemic impact on purine, neurotransmitter, and vitamin B metabolism evident throughout the upper respiratory, gut, and circulating metabolomes. These pathways can be connected through phosphoribosyl pyrophosphate (PRPP), a metabolite in the pentose phosphate pathway (PPP). PRPP is a biochemical intermediate for purines, pyrimidines, B vitamins, and tryptophan [84,85]. The PPP is vital for maintaining cellular reducing capacity, and metabolism can be rerouted to increase flux through the PPP in response to oxidative stress [86]. This increased flux through the PPP has previously been described in response to nicotine [87]. A potential mechanism underlying the systemic impact on purine, neurotransmitter, and vitamin B metabolism could be the rerouting of cellular metabolism via increased flux through the PPP in an effort to combat vaping-induced oxidative stress to generate antioxidants [88].

To understand differences between the pathophysiological impact of vaping and combustible smoking (tobacco/marijuana), metabolite alterations likely associated with vaping were identified in EBC, gargle, plasma, and stool samples. Surprisingly, the gargle samples displayed the fewest metabolite differences unique to participants who vape as only three metabolites (sucralose, glutathione, and riboflavin) were uniquely altered by vaping. Riboflavin is essential in the conversion of glutathione disulfide to its reduced form, glutathione, which is a powerful antioxidant [89]. Riboflavin is also required for methylation [90]. Combined, this may indicate metabolic reprogramming induced by vaping, though further research is needed to confirm. Similarly, only five metabolites were unique to participants who vape in EBC: uric acid, methionine, 5-HIAA, methionine sulfoxide, and d-gluconate. Methionine is an essential amino acid for sulfur metabolism, methylation reactions, redox metabolism, protein synthesis, and cellular signaling. However, under oxidative stress, methionine is oxidized to methionine sulfoxide, thereby impairing these pathways [91,92]. These data suggest that vaping alters methionine metabolism to a greater extent than combustible smoking.

In the stool metabolome, there were 25 metabolites exclusively altered by vaping. Allantoate, sulfolactate, histamine, glutathione, indole, shikimate, tryptophan, folate, allantoin, and phenylalanine were among the metabolites altered only by vaping, demonstrating a significant impact on neurotransmitter, purine, and redox metabolism. Metabolites in circulation impacted by vaping but not combustible smoking were glutathione disulfide, pantothenate, hypoxanthine, phenylalanine, indole, and tryptophan, again revealing a significant impact on neurotransmitter metabolism.

Surprisingly, neurotransmitter metabolism was a unique and systemic signature of vaping, highlighting the impact on the gut–brain axis. This finding was alarming because vaping is an emerging public health crisis among adolescents and young adults due to the high prevalence of vaping in this population [93]. While it is well recognized that nicotine is harmful for developing brains, it is often overlooked that many e-cigarettes contain higher concentrations of nicotine than combustible cigarettes [94]. The human brain is not fully developed until ~26 years of age [95], making vaping especially detrimental to the developing brains among adolescents and young adults. Within this population, vaping can result in cognitive deficits, impaired in memory, and executive dysfunction leading to impulse control, addiction, and mood disorders [94]. Although the long-term impact is unknown, these data provide additional evidence that vaping is not risk free as demonstrated by the impact of vaping on the gut–brain axis and neurotransmitter metabolism. This is a significant finding supporting the urgent need for public health initiatives to educate communities on the potential neurological risks associated with vaping.

### 4.5. Limitations

Our study has some limitations that should be considered when interpreting the findings. First, the sample size was relatively small (n = 23), which may limit the potential to generalize our results to a larger population. Due to the small number and the variety of substances/devices, it was hard to group the individuals who smoked into homogeneous groups. As a result, we were not able to separate the cohort into vaping exclusively (without combustible smoking), combustible tobacco smoking exclusively, or exclusive combustible marijuana smoking. Instead, we used metabolites that were differentially abundant between participants who vape and the control cohort but that were not differentially abundant between participants who smoke combustibles and the control cohort to identify metabolites altered uniquely by vaping. However, it is also possible that these signatures could be a dose-dependent response to nicotine since the vaping cohort also smoked cigarettes, although there was not a significant difference in the number of cigarettes smoked between the cohorts. To address this, we propose an experiment controlling nicotine use so both cohorts intake the same amount of nicotine. Additionally, the data were collected through self-reported questionnaires, introducing the potential for response bias. The cross-sectional design also restricts our ability to make causal inferences about the relationships observed. This study was designed to investigate vaping-associated metabolic changes, so changes attributed to combustible tobacco and marijuana smoking were only briefly mentioned.

## 5. Conclusions

In conclusion, the respiratory, plasma, and intestinal metabolomes of PWH who vape, PWH who smoke combustible tobacco/marijuana, and PWH controls were analyzed. This untargeted, hypothesis-generating analysis revealed that vaping results in systemic metabolic dyshomeostasis, specifically in purine, neurotransmitter, vitamin B, and energy metabolism. In addition, these data provided the first evidence that DHPS, a microbial metabolite with an unknown role in human physiology, may be linked to vaping and smoking-induced metabolic dyshomeostasis and provides a basis for future research investigating the role of DHPS in human health. Future studies could benefit from longitudinal sampling including lipid analyses to evaluate the effect of vaping on the lipidome, oral and stool microbiome analysis, and a targeted metabolomics analysis covering nicotine degradation. We encourage further research in this area to understand the full biological and clinical significance of these changes.

## Figures and Tables

**Table 1:** Demographic information and sample characteristics.

Variable	Control Group (n = 10)	Participants Who Smoke Combustible Tobacco and/or Marijuana (n = 7)	Participants Who Use E-Cigarettes/Vape (n = 6)
Age (years)	51 (SD= 7.33)	48.83 (SD = 14.14)	43.83 (SD = 11.92)
Male	6 (60%)	6 (85.71%)	5 (83.33%)
Female	4 (40%)	1 (14.28%)	1 (16.66%)
Ethnicity			
African-American	7 (70%)	5 (71.42%)	3 (50%)
Caucasian/White	1 (10%)	1 (14.28%)	3 (50%)
Hispanic/Latino	1 (10%)	-	-
Did not report	1 (10%)	1 (14.28%)	-
BMI	33.84 (SD = 6.56)	40.53 (SD = 14.06)	28.52 (SD = 5.93)

Data are represented by mean and standard deviation (SD) or the absolute number of subjects and the percentage they represent in the group.

**Table 2:** Substance use statistics of the groups.

Variable	Control Group (n = 10)	Participants Who Smoke Combustible Tobacco and/or Marijuana (n = 7)	Participants Who Smoke E-Cigarettes/Vape (n = 6)
Tobacco smoking			
Number of cigarettes ^1^	0	4.83 (SD = 4.57)	6.5 (SD = 8.8)
* Smoking Years *	-	16.8 (SD = 13.92)	24.33 (SD = 13.93)
* E-cigarettes use (days) * ^1^	-	-	20.33 (SD = 14.97)
Marijuana * Lifetime (yes) *	6 (60%)	7 (100%)	6 (100%)
* Days of use * ^1^	0	6.33 (SD = 11.75)	21.5 (SD = 13.47)
Alcohol use			
Lifetime (yes)	8 (75%)	7 (100%)	6 (100%)
* Days of use * ^1^	8 (SD = 4)	2 (SD = 0)	2 (SD = 0)

Data are represented by mean and standard deviation or the absolute number of subjects and the percentage they represent in the group. ^1^ Recent use in last 30 days.

## Data Availability

The raw data supporting the conclusions of this article will be made available by the authors on request.

## Supplementary Materials

The following supporting information can be downloaded at: https://www.mdpi.com/article/10.3390/metabo14080434/s1, Figure S1: 3D PLS-DAs of vaping-induced global metabolome alterations; Figure S2: Exhaled breath condensate metabolome alterations from combustible smoking; Figure S3: Serum metabolome; Figure S4: VIP scores for plasma; Figure S5: Impact of combustible smoking on plasma EDTA; Figure S6: Impact of combustible smoking on plasma heparin; Figure S7: Stool metabolites correlated with DHPS; Table S1: Metabolites unique to vaping.
