# Supplementary material for: Effects of E-Cigarettes on the Lung and Systemic Metabolome in People with HIV
Source: Metabolites. 2024 Aug 6;14(8):434. doi: 10.3390/metabo14080434 (PMC11356516; doi:10.3390/metabo14080434)
Supplement: Supplementary file 1 [file metabolites-14-00434-s001.zip › metabolites-3084224-supplementary figures and tables.pdf]

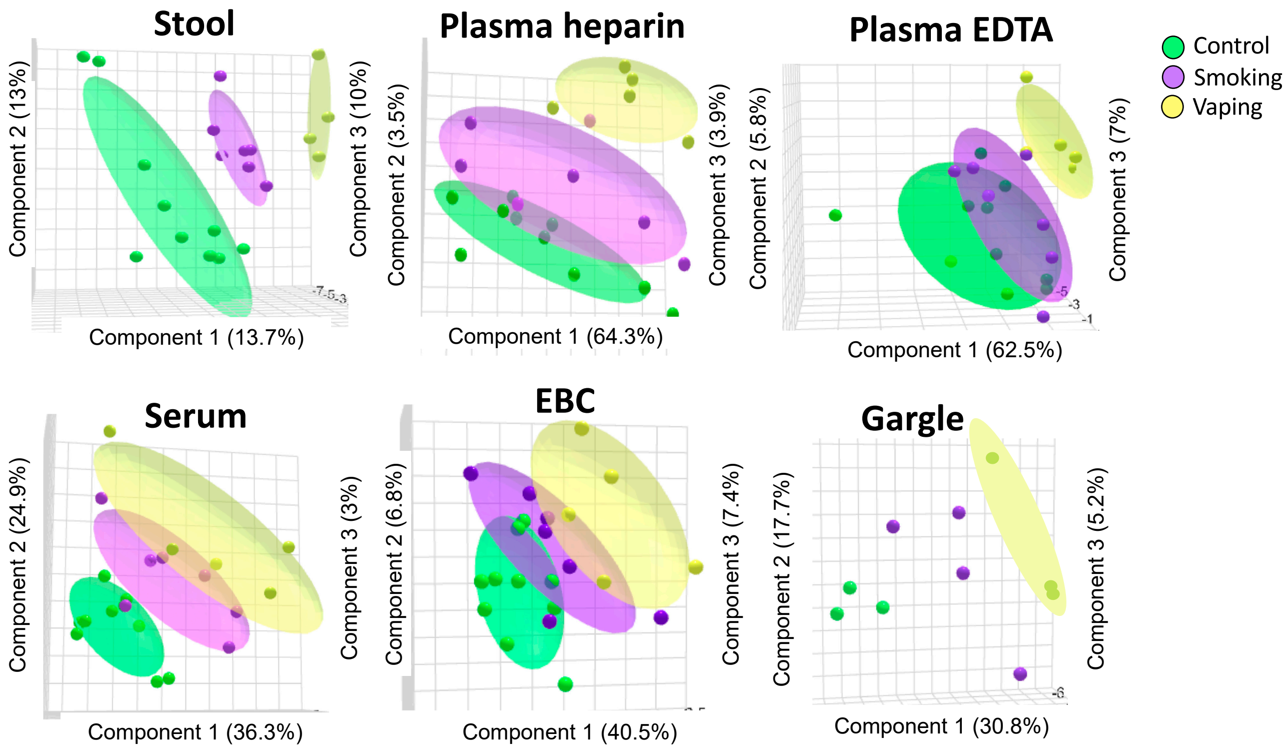

Figure S1. Vaping results in dramatic alterations to the oral, stool, and plasma metabolomes. 3D Partial Least Squares Discriminant Analysis (PLS-DA) comparing the metabolic profiles of participants who vape “vaping” (yellow), participants who do not vape “smoking” (purple), and controls (green). The smoking cohort (purple) included participants who combustible tobacco/marijuana smoke. EBC, exhaled breath condensate.

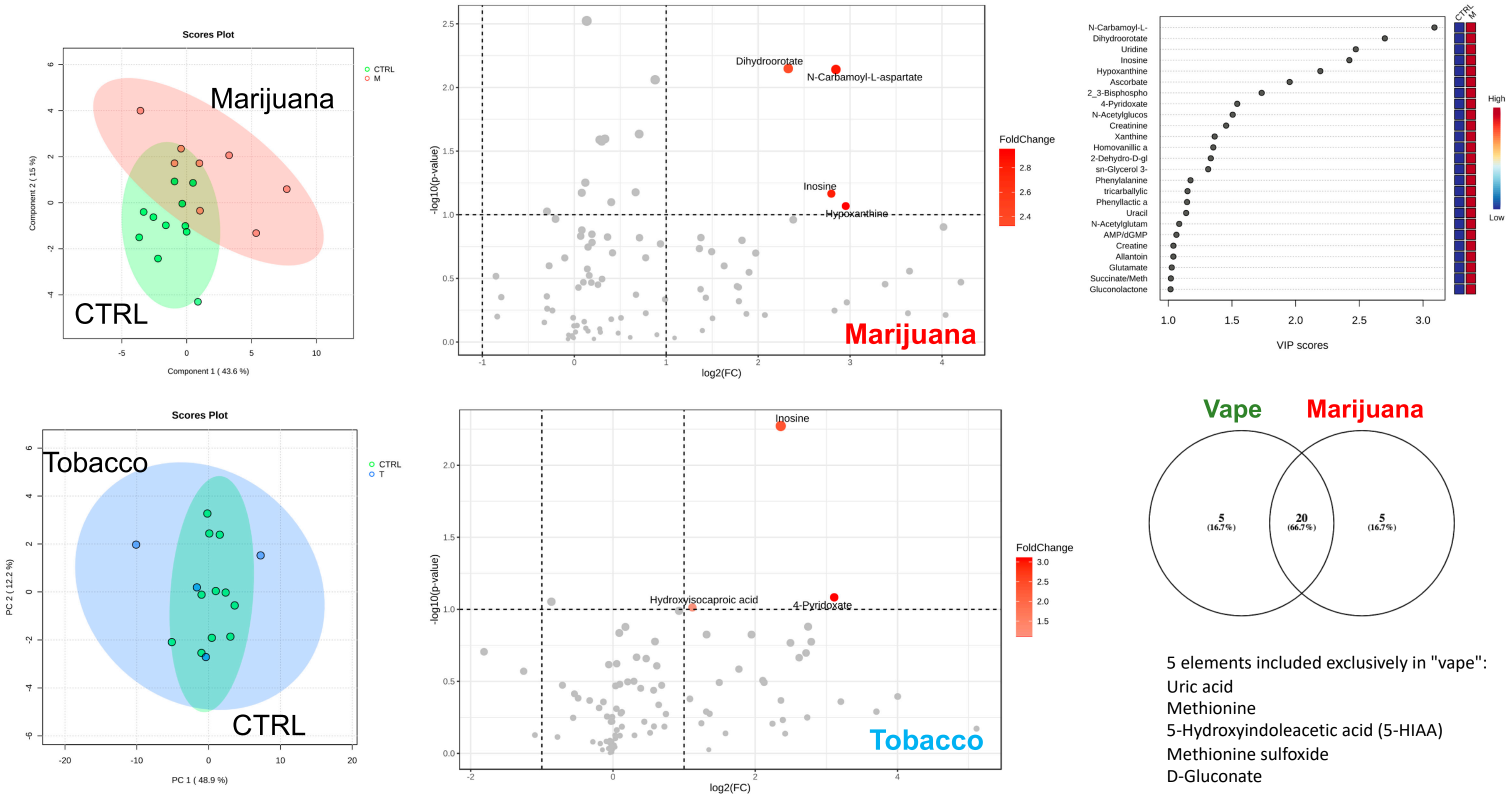

Figure S2. Combustible marijuana and tobacco smoking impact on exhaled breath condensate metabolome. Partial Least Squares Discriminant Analysis (PLS-DA) were used to assess the impact on the global metabolome. Each metabolite was assigned a variable importance in projection (VIP) score (top right) to assess its contribution to the differences in metabolic profiles. The 15 metabolites with the highest VIP scores were shown. A VIP score >1 indicates the metabolite is a significant driver of the observed separation between groups. Volcano plots were used to visualize metabolites altered in each cohort. The x –axis displays the $\log_2$ fold change and the y-axis displays significance. VIP scores were not analyzed for the Tobacco vs control cohort since the groups overlap. Metabolites with a VIP score >1 for each pairwise comparison were used to identify metabolites altered only be vaping. The Venn diagram (bottom right) comparing these metabolites shows 5 unique markers for vaping.

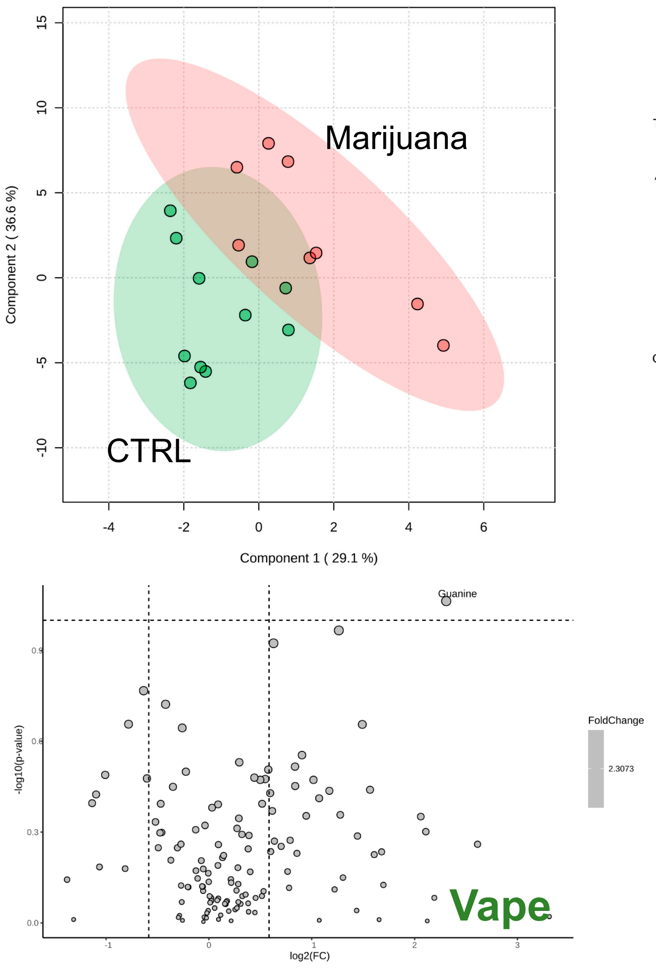

Figure S3. Vaping and combustible marijuana smoking impact on serum metabolome. Partial Least Squares Discriminant Analysis (PLS-DA) were used to assess the impact on the global metabolome. Each metabolite was assigned a variable importance in projection (VIP) score (top right) to assess its contribution to the differences in metabolic profiles. The 15 metabolites with the highest VIP scores were shown. A VIP score >1 indicates the metabolite is a significant driver of the observed separation between groups. Volcano plots were used to visualize metabolites altered in each cohort. The x –axis displays the log_2 fold change and the y-axis displays significance.

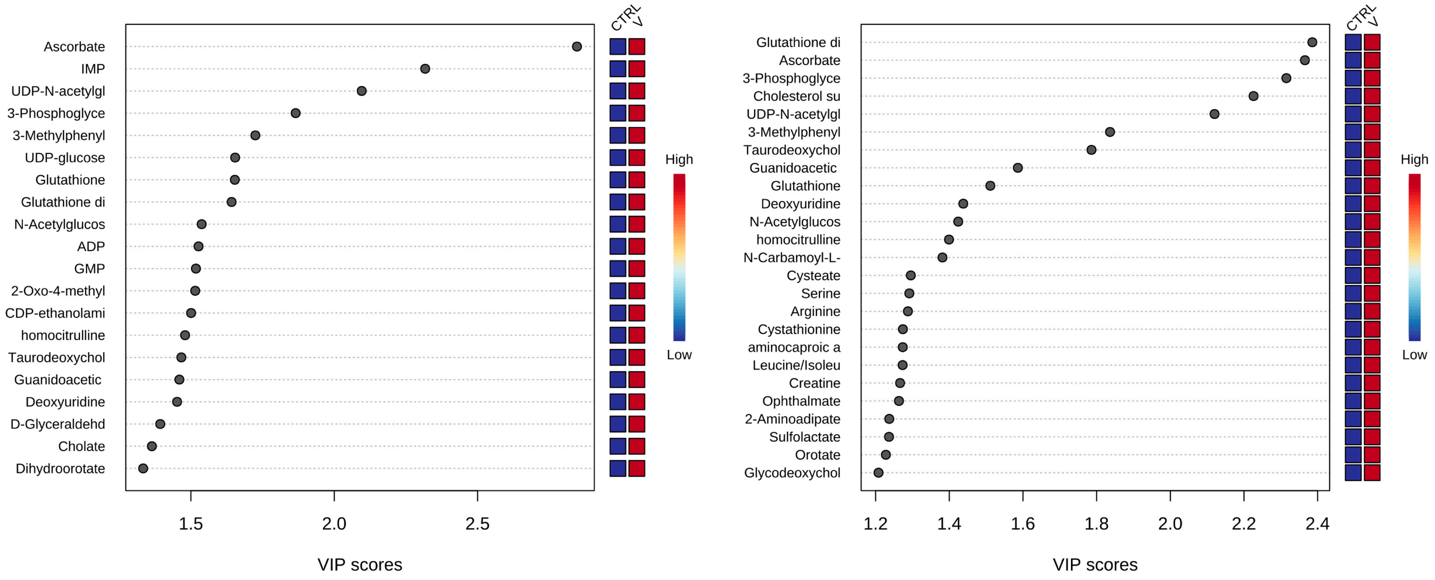

Figure S4. Top 15 metabolites driving metabolic profile differences induces by vaping in plasma EDTA (left) and plasma heparin (right)**.** Each metabolite was assigned a variable importance in projection (VIP) score to assess its contribution to the differences in metabolic profiles in the PLS-DA model. The 15 metabolites with the highest VIP scores were shown. A VIP score >1 indicates the metabolite is a significant driver of the observed separation between groups.

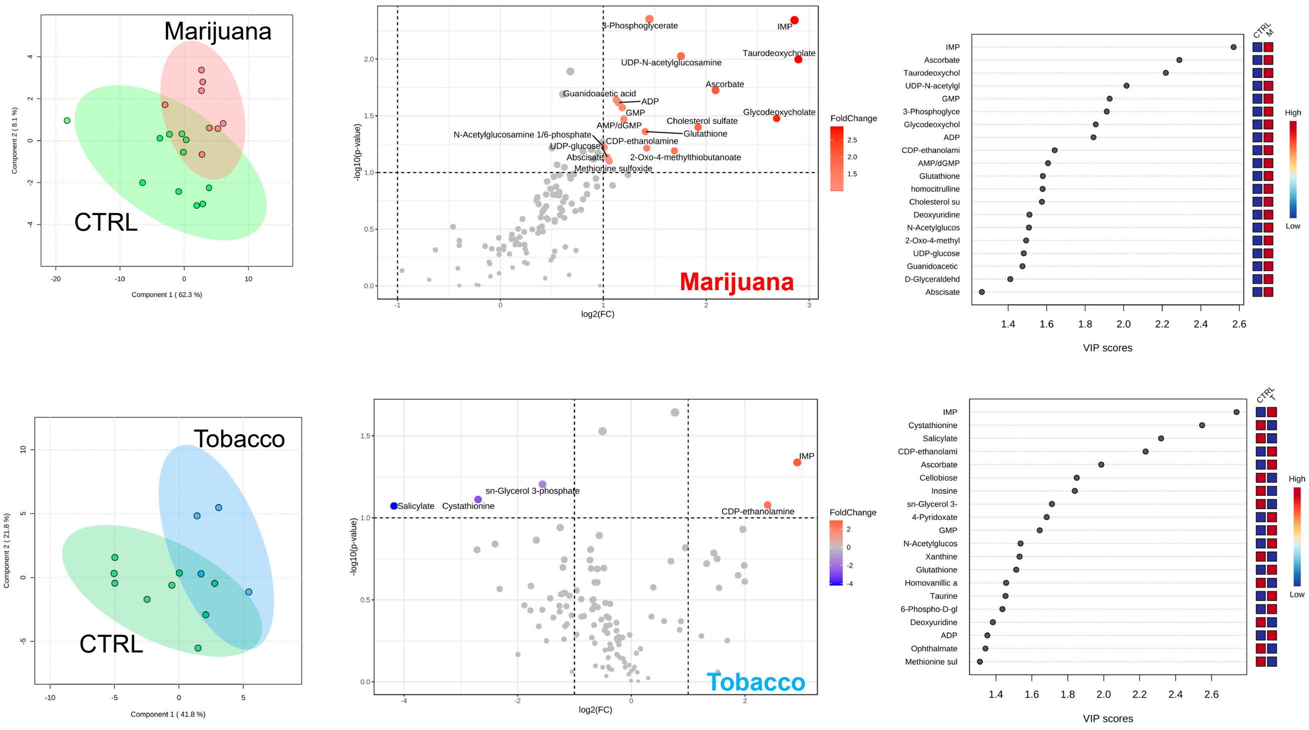

Figure S5. Combustible marijuana and tobacco smoking impact on plasma EDTA metabolome. Partial Least Squares Discriminant Analysis (PLS-DA) were used to assess the impact on the global metabolome. Each metabolite was assigned a variable importance in projection (VIP) score to assess its contribution to the differences in metabolic profiles. The 15 metabolites with the highest VIP scores were shown. A VIP score >1 indicates the metabolite is a significant driver of the observed separation between groups. Volcano plots were used to visualize metabolites altered in each cohort. The x –axis displays the log_2 fold change and the y-axis displays significance.

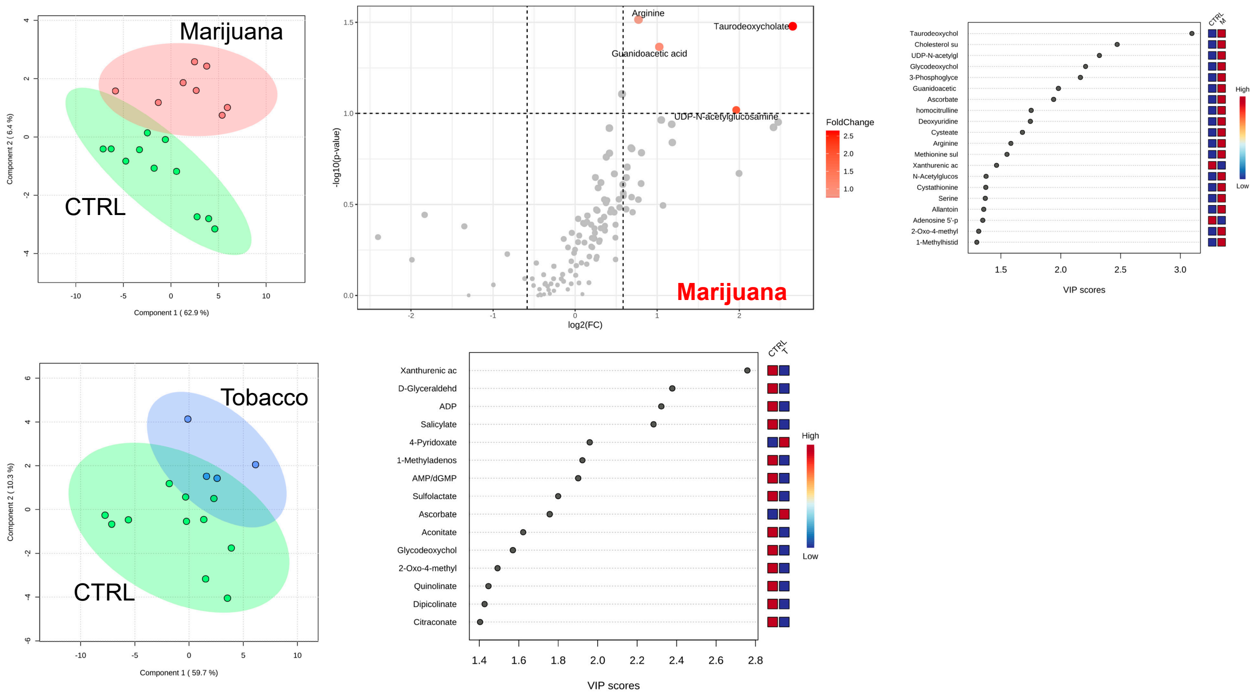

Figure S6. Combustible marijuana and tobacco smoking impact on plasma heparin metabolome. Partial Least Squares Discriminant Analysis (PLS-DA) were used to assess the impact on the global metabolome. Each metabolite was assigned a variable importance in projection (VIP) score to assess its contribution to the differences in metabolic profiles. The 15 metabolites with the highest VIP scores were shown. A VIP score >1 indicates the metabolite is a significant driver of the observed separation between groups. Volcano plots were used to visualize metabolites altered in each cohort. The x –axis displays the log_2 fold change and the y-axis displays significance.

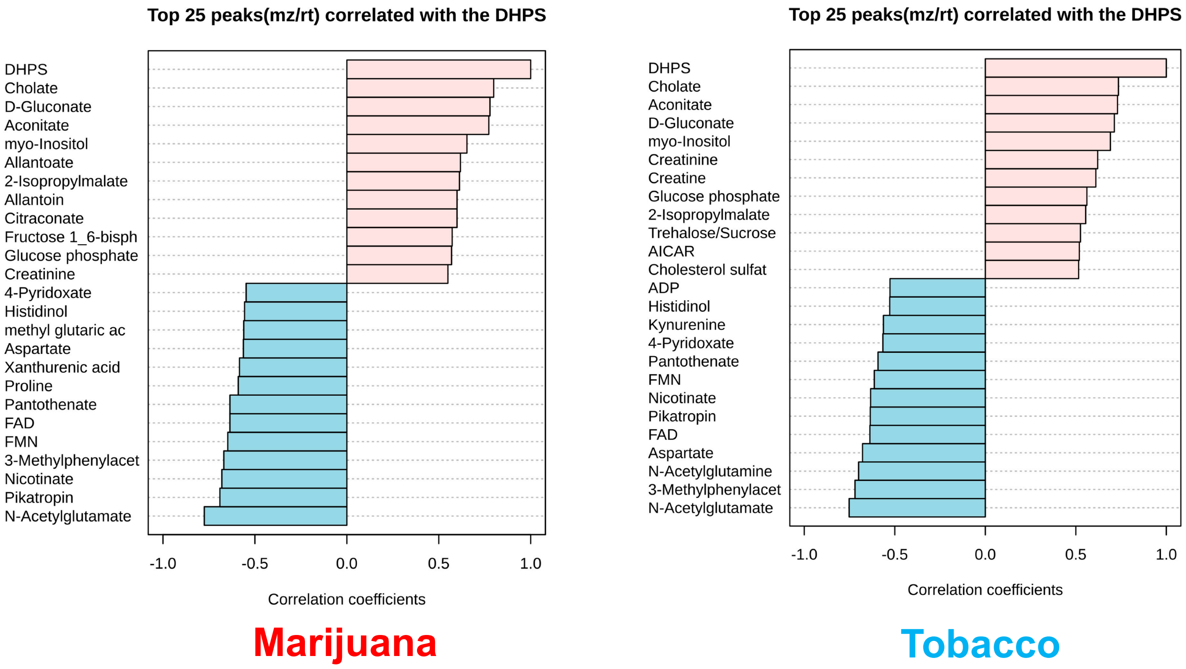

Figure S7. Stool metabolites correlated with DHPS in combustible marijuana and tobacco smoking cohorts using Pearson's r correlation coefficients. DHPS, 2,3-dihydroxypropane-1-sulfonate
